# Mixed-methods evaluation of an enhanced asthma biologics clinical pathway in the West Midlands UK

**DOI:** 10.1038/s41533-024-00365-y

**Published:** 2024-05-01

**Authors:** Sarah Damery, Janet Jones, Elfatih Idris, Angela Cooper, Holly Minshall, Chris Clowes, Kate Jolly

**Affiliations:** 1 Institute of Applied Health Research, University of Birmingham, Edgbaston, West Midlands B15 2TT UK; 2 Royal Stoke University Hospital, University Hospitals of North Midlands NHS Trust, Newcastle Road, Staffordshire, ST4 6QG UK; 3 Health Innovation West Midlands, Faraday Wharf, Holt Street, Birmingham, B7 4BB UK

**Keywords:** Asthma, Diagnosis

## Abstract

Biologic treatments can alleviate severe asthma symptoms and reduce health service use. However, service capacity limits and low referral rates from primary care indicate unmet patient need. We report a mixed-methods evaluation of an enhanced severe asthma pathway implemented in Staffordshire and Stoke-on-Trent, UK which aimed to optimise primary care referrals through training/education, and increased capacity in specialist clinics. Quantitative analysis assessed patient wait times between pathway stages, prescribing changes, exacerbations, hospital admissions and asthma control. Interviews with 12 stakeholders evaluated perceptions of the enhanced pathway across settings. In 12 months, 564 patients from 28 general practices were reviewed for biologics eligibility, of whom 125 (22.2%) were referred for specialist assessment. Wait times were significantly lower under the enhanced pathway when compared against historic patients following the standard pathway, and reduced overall from a mean of 76.4 to 26.7 weeks between referral and biologics initiation (*p* < 0.001). Patients commencing biologics (*n* = 46) showed significantly reduced reliever inhaler prescribing rates (*p* = 0.037), 60% lower oral steroid use (*p* < 0.001), significantly reduced exacerbation rates (*p* < 0.001) and fewer hospital admissions (*p* < 0.001) compared with the 12 months pre-treatment. Mean asthma control scores reduced from 3.13 pre-initiation to 1.89 post-initiation (*p* < 0.001) – a clinically significant improvement. Interviewees viewed the enhanced pathway positively, although ongoing issues related to difficulties engaging primary care amid concerns around increased workloads and pathway capacity. The large number of referrals generated from a comparatively small number of general practices confirms substantial unmet need that an enhanced severe asthma pathway could help address if implemented routinely.

## Introduction

Uncontrolled severe asthma is associated with reduced patient quality of life^1^ and significantly impacts National Health Service (NHS) resources through hospital admissions, Accident and Emergency (A&E) use, and prescribing of inhaled or oral corticosteroids (OCS) and short-acting beta agonists (SABA)^2^. Patients with uncontrolled severe asthma have an eight-fold mortality risk^3^, and account for around 60% of total asthma costs despite only comprising around 5% of patients with asthma^4^. Several severe asthma sub-types can be treated effectively by asthma biologics, which use monoclonal antibodies to target specific cells to stop the processes that cause lung inflammation. These treatments can allow OCS use to be reduced or stopped, improve symptom management, and reduce dependence on NHS services^2,5^.

Consensus guidelines recommend that patients with severe asthma should be systematically evaluated and have their diagnosis confirmed by a dedicated multi-disciplinary team (MDT) with clinical expertise in severe asthma assessment and management^6^. However, despite their effectiveness, biologic treatments have strict eligibility criteria, in large part due to their high cost^7^. Imprecise referral criteria can result in delays in referring patients to a specialist asthma centre from primary care^8^, under-diagnosis, or inappropriate referrals of patients later found to be ineligible^9,10^. A review of patient journey times to asthma biologics showed that more than 80% of patients live with uncontrolled asthma for over a year before referral to specialist asthma services; experience a median wait of 13 weeks between referral and specialist review, and six weeks from biologics approval to initiation^11^. These waiting times are also subject to significant regional variation^12^.

The area of Staffordshire and Stoke-on-Trent in the West Midlands, UK has a higher than average prevalence of severe asthma. Data modelling and local sub-analysis extrapolating information from the NHS Quality Outcomes Framework (QoF) for 2019/20 suggests substantial unmet need for severe asthma management within the catchment^13^. Estimates indicate there may be around 450 patients in the region eligible for asthma biologics, yet as of 2021, just 65 patients were receiving treatment. Barriers to the widespread use of biologic treatments in the area included: low rates of patient identification and onward referral from primary care; capacity limits on patient assessment by multidisciplinary teams (MDT) within the local and regional severe asthma service; significant geographical distance to regional tertiary referral centre, and low uptake of asthma biologics self-administration by eligible patients.

Consequently, as part of a national programme funded by the NHS Accelerated Access Collaborative (AAC) Rapid Uptake Product (RUP) programme, an enhanced severe asthma pathway was implemented in Staffordshire and Stoke-on-Trent from November 2021, aimed at optimising patient referrals from primary care, increasing clinic capacity for MDT assessments, and expanding the use of home care and self-administration of biologics by eligible patients. This paper reports the findings of an independent, mixed methods evaluation of the effectiveness of the pathway changes in improving patient referrals, treatment waiting times and clinical outcomes.

## Results

### Primary care referrals and patient eligibility for biologics

By the end of October 2022, 28 GP practices had been visited by the nurse educator and the SPECTRA tool run on their systems to identify patients who may be potentially eligible for biologics treatment. Across these practices, 564 patients were reviewed for eligibility, and 125 were referred to the biologics clinic in secondary care for further assessment (22.2%). By November 2022, 87 of these 125 patients had either started on asthma biologics, or were scheduled to start by the end of the year (69.6%). A total of 64/87 were (or would be once initiated) self-administering their treatments through the home care service (73.6%). The reasons that 38/125 patients did not start biologics during the study period were: ineligibility (*n* = 21), eligibility decision still awaited (*n* = 8), patient non-adherence with appointments (*n* = 4), patient declined biologics (*n* = 3), and patient diagnosed with Chronic Obstructive Pulmonary Disease (COPD) (*n* = 2).

### Patient waiting times

When wait times for discrete stages of the severe asthma pathway were compared for the ‘standard’ pathway (historic patients referred from primary care before between 11/2020 and 11/2021, *n* = 37) vs. the enhanced pathway (patients referred from primary care from 11/2021 onwards, *n* = 143), the time patients spent at each pathway stage were substantially lower within the enhanced pathway compared to the standard pathway (Table 1). The mean wait between referral and first secondary care appointment was 59% shorter under the enhanced pathway compared to the standard pathway (10.7 weeks vs. 21.2 weeks; *p* = 0.002). The time from the first appointment in secondary care to first secondary care follow-up appointment and/or severe asthma diagnosis was shorter by 45% under the enhanced pathway. The time from follow-up/diagnosis and MDT discussion showed mean waiting times under the enhanced pathway of 6.7 weeks, compared with 17.9 weeks under the standard pathway (a 63% reduction; *p* = 0.004). Although wait times between MDT meeting and biologics initiation reduced by around half (from 15.9 to 8.0 weeks), this was not statistically significant. Taking the pathway from referral to biologics initiation as a whole, mean waiting time was 76.4 weeks for the standard pathway, compared with 26.7 weeks for the enhanced pathway (*p* < 0.001).

**Table 1:** Comparison of severe asthma pathway waiting times between patient cohorts.

Pathway stage	Historic patients (*n* = 37)	Current patients (*n* = 143)	Significance	Mean change
	Mean weeks (range)	Mean weeks (range)	*p*-value	% change in waiting time
Stage 1: Referral to first appointment in secondary care	21.2 (1.0–74.3)	8.5 (1.0–26.1)	*p* = 0.002	59% reduction
Stage 2: First appointment secondary care to follow-up/diagnosis	24.3 (6.0–67.7)	13.3 (1.0–45.1)	*p* < 0.001	45% reduction
Stage 3: Follow-up/diagnosis to MDT discussion	17.9 (2.0–40.8)	6.7 (1.0–23.3)	*p* = 0.004	63% reduction
Stage 4: MDT discussion to biologics initiation	15.9 (4.0–99.9)	8.0 (3.0–16.9)	*p* = 0.063	49% reduction (not statistically significant)
Overall: Pathway from referral to treatment initiation	76.4 (23.0–134.0)	26.7 (16.0–53.8)	*p* < 0.001	65% reduction

### Clinical outcomes

Of the 87 patients in the cohort deemed eligible for biologics treatment under the enhanced pathway, 41 were excluded as incomplete cases (19 patients were yet to begin treatment; 22 had fewer than three months of follow-up data). This left 46 patients with pre- and post-biologics initiation data for comparison. The mean length of follow-up for patients following the enhanced pathway was 6.3 months.

Data demonstrated significant post-initiation improvements in key service and patient-related outcomes (Table 2). Inhaled corticosteroid prescribing rates increased non-significantly by 8% from a mean of 1.37 per patient per month to 1.48 (*p* = 0.210). Rates of SABA prescribing significantly reduced (*p* = 0.037), from a mean of 0.93 to 0.76 per month. Oral steroid prescribing reduced by 60%, from a pre-biologics mean of 0.37 per month to a post-treatment monthly mean of 0.12 (*p* < 0.001). The mean monthly rate of asthma-related exacerbations experienced by patients after starting biologic treatments significantly reduced from 0.36 to 0.13 (*p* < 0.001). Rates of hospital admission (although small in number both before and after biologics initiation) also reduced significantly, from 0.12 to 0.01 (*p* < 0.001). ACQ6 scores, denoting changes over time in symptom control, showed a statistically and clinically significant improvement for patients starting biologics. In the pre-biologics period, the mean ACQ6 score was 3.13. This reduced to 1.89 after biologics initiation, significant to the *p* < 0.001 level. Across all patients with a pre- and post-biologics ACQ6 score, the average difference over time was a reduction of 1.2 points (range −4.8 to +1.1), with 27 patients (62.8%) improving their ACQ6 score over time by the clinically important minimum difference of 0.5 points.

**Table 2:** Clinical outcomes for patients initiating biologics treatment under the enhanced pathway.

	Pre-biologics		Post-biologics			
	Mean rate/month (SD)	Range	Mean rate/month (SD)	Range	% change in mean rate	*p*-value
PRESCRIBING						
Inhaled corticosteroids	1.37 (0.55)	0.5–2.3	1.48 (0.52)	0.4–2.9	+8%	*p* = 0.210
Short-acting beta agonists	0.93 (0.46)	0.2–2.2	0.76 (0.51)	0.0–2.2	−18%	*p* = 0.037
Oral corticosteroids	0.37 (0.37)	0.0–1.3	0.12 (0.22)	0.0–0.8	−60%	*p* < 0.001
EXACERBATIONS						
Exacerbations	0.36 (0.26)	0.0–1.0	0.13 (0.22)	0.0–0.8	−64%	*p* < 0.001
HOSPITAL USE						
Admissions	0.12 (0.16)	0.0–0.8	0.01 (0.36)	0.0–0.2	−92%	*p* < 0.001
ASTHMA CONTROL	Mean score (SD)		Mean score (SD)			
ACQ6 scores	3.31 (1.34)	0.6–5.4	1.89 (1.36)	0.0–5.0	40% improvement	*p* < 0.001

### Qualitative data

A total of 12 semi-structured interviews were carried out with key stakeholders (respiratory consultants (*n* = 3), respiratory nurses (*n* = 3), GPs (*n* = 1), pharmacists (*n* = 1), practice nurses (*n* = 1), project managers (*n* = 2) and directorate managers (*n* = 1)). Headline qualitative findings are summarised below, with detailed mapping of data against the CFIR domains shown in Table 3.

**Table 3:** Qualitative data mapped onto the Consolidated Framework for Intervention Research (CFIR) framework.

Domain of CFIR framework	Summary of relevant qualitative data
Intervention characteristics (advantages, design, adaptability, complexity)	•Reduced time from referral into secondary care and initiation of biologics treatment •More appropriate referrals being made by primary care •Introduction of SPECTRA tool effective in identifying appropriate patients for referral •Well-received training and education for staff working in primary care to improve awareness of severe asthma management •Improved and more timely access to biologic treatments for eligible patients •Facilitating GP engagement with the enhanced pathway was sometimes challenging •Financial cost, ensuring adequate staffing and time were available across the pathway was considered important •Concern that the enhanced pathway would entail additional workloads in primary care
‘Outer setting’ – wider links required for effective intervention	•Additional asthma follow-up clinic introduced so the secondary care team could ensure timely diagnostic testing and follow-up •Concern by some primary care providers about using a software tool (SPECTRA) on their practice systems that had been developed by a pharmaceutical company •Good communication noted between community teams, primary care and teams in the acute setting
‘Inner setting’ – influence of culture, norms, relative priority of intervention	•Nurse educator role working with primary care was considered important to improve integration and build positive relationships between primary care and secondary care service providers •Primary care is required to provide the same level of care for all long term conditions and cannot single out asthma for special consideration given resource constraints
Individual characteristics (knowledge, beliefs about intervention, individual use, self-efficacy)	•Positive views about the effectiveness of the enhanced pathway and the clarity provided for primary care regarding referral criteria •Expedited process for assessing patient eligibility and clear pathway towards treatment initiation •Enthusiasm from participants across all settings about the enhanced pathway and its potential •Too soon to tell if primary care providers would be confident and have the necessary resources to deliver the pathway in the longer-term once the nurse educator was no longer available
Implementation process (planning, execution, reflecting)	•Participants raised concerns about sustainability •Too soon to know the longer-term outcomes of the enhanced pathway •A slower paced rollout was recommended to allow primary care practices to fully embed the pathway and encourage ‘ownership’ •Complexity and potential for confusion with enhanced pathway operating alongside elements of the standard pathway such as the ‘choose and book’ system

### Benefits of the enhanced severe asthma pathway

All interview participants highlighted multiple benefits of the enhanced pathway for patients and staff. The standard severe asthma pathway was felt to be disjointed, with many patients with severe and difficult asthma managed in primary care without being referred to specialist services:

*“I think we have failings throughout the whole of the pathway, and that is the responsibility of both primary care and secondary care. It’s a mess.”* (Respiratory consultant, secondary care)

Interviewees felt that the time between patient referral from primary care and the first appointment at the severe asthma clinic in secondary care had been shortened considerably by the enhanced pathway. Members of the secondary care respiratory team felt that the enhanced pathway had also improved the proportion of patients appropriately referred into secondary care for assessment, although potential future capacity issues were recognised associated with a greater number of referrals being made:

“*I think one of the positives, because of the way the clinic was set up is you didn’t get caught up in the whole waiting list system for secondary care, but I think that is going to become more of a problem as you refer more patients in.”* (General practitioner, primary care)

A significant strength of the enhanced pathway was agreed by several participants to be the proactive element in a nurse educator supporting general practices to identify patients who may be eligible for biologic treatments by working closely with the practices and searching practice lists:

*“So this project is absolutely amazing – what it’s doing is it proactively goes out to the GP practices, and – I am going to use the word in a friendly term – hunts down patients. So they are really out to scoop up those patients and refer them to secondary care.”* (Operational manager, non-clinical)

The training and education component of the enhanced pathway was favourably received by those working in primary care, and was felt to be effective in helping to identify patients with severe asthma who would benefit from specialist assessment:

“*So I think certainly the education that’s been provided in primary care now is highlighting that need, that unmet need if you like…they get an appointment quicker than they would have done if they had gone through the traditional route.”* (Asthma nurse, secondary care)

### Challenges associated with enhanced pathway implementation

The participation of primary care in the enhanced pathway was recognised as a challenge, and there were difficulties engaging primary care providers in the project. This was particularly due to concerns over time and staff resources, and worries that focusing resources on identifying patients for referral could entail increased workloads for over-stretched practice staff:

*“First of all, is we’re only able to target some GP practices, and there are far more practices who are eligible for us to get involved with…some practices are not necessarily welcoming, some practices are welcoming but we don’t have the nurse resource to actually go and do the digging.”* (Respiratory consultant, secondary care)

Interviewees from some primary care practices also expressed reluctance to use the SPECTRA software tool on their practice systems because this has been developed by a pharmaceutical company. Furthermore, several interviewees noted that administration and co-ordination of the enhanced pathway, although straightforward logistically, was more time-consuming than the standard pathway because elements of the standard and enhanced pathways were running in parallel rather than one having replaced the other. This was particularly evident in relation to the ongoing maintenance of the standard ‘Choose and Book’ system for facilitating patient referrals from primary care, which was perceived as creating an unhelpful two-tier referral system for severe asthma. Participants from primary care also highlighted that asthma management was just one of the clinical areas that needed attention, and that handling competing priorities was an ongoing challenge:

*“Because what we are doing with respiratory, we’ve got to do with diabetes, we’ve got to do with heart disease, we have to do everything at the same level these days…there’s only so much we can do.”* (Advanced Nurse Practitioner, primary care)

Finally, the ongoing sustainability of the severe asthma pathway was highlighted by a number of interview participants. This was seen as important both in terms of the need for greater ‘ownership’ of the pathway by primary care providers once the resource of the nurse educator was no longer available and it became the sole responsibility of practices to proactively identify patients for referral, and the need for staff resources across the pathway to ensure that it could continue to operate effectively once the targeted funding that allowed it to be introduced had ceased:

“*So, I think that our worry is more around sustainability, and if there are opportunities to create the service in terms of nursing staff, physicians etc., that would be wonderful.”* (Respiratory consultant, secondary care)

## Discussion

Increasing capacity at a secondary care severe asthma clinic and providing training and education on appropriate diagnosis and referral in primary care, alongside implementation of a nurse educator to work with primary care providers to identify eligible patients was associated with substantially reduced patient waiting times for referral, diagnostic confirmation and initiation on asthma biologics. A total of 87/125 referred patients were confirmed eligible for biologics treatment at the time the evaluation ended, demonstrating improved appropriateness of referral from primary care and a ‘hit rate’ of nearly 70% of patients referred from primary care going on to be approved for biologic treatments. Patients who initiated biologics during the evaluation and had at least three months follow-up data (*n* = 46) experienced significant reductions in SABA and inhaled/oral steroid prescribing, and significant reductions in asthma-related exacerbations and hospital admissions. Patients also saw significant improvements in asthma control when pre- and post-biologics ACQ6 data were compared.

Qualitative data demonstrated that training and education in primary care was effective, and the enhanced pathway was associated with improved efficiency across healthcare settings, with a streamlined process of referring patients from primary care to specialist services. The increased capacity within the severe asthma clinic and additional MDT meant that patients could be assessed quickly and initiated on biologics as appropriate. However, it was difficult to engage some primary care practices, and there was concern from primary care that focusing on severe asthma management would entail increased workload for practice staff and compete with resources available for managing other long term conditions. There was also some reluctance by practice staff to use a software tool on their systems that had been developed by a pharmaceutical company. A two-tier referral system remained despite the introduction of the enhanced pathway, which was felt to have introduced complexity to the system. Finally, there were some concerns about the ongoing sustainability of the enhanced pathway once targeted funding for its implementation ended. It is clear that core resource from the local Integrated Care Board (ICB) would be required across primary and secondary care to consolidate the additional staff and clinic capacity that allowed the enhanced severe asthma pathway to: a) uncover substantial unmet need for severe asthma services in primary care, and b) expedite patient progression through the pathway by substantially reducing waiting times for specialist service assessment and biologics initiation for those deemed eligible. Despite its effectiveness, the pathway outlined in this study may not be sustainable because of the multiple clinical priorities that ICBs in the NHS are required to manage in the face of increasingly restricted financial resources.

This study was undertaken in recognition of a national need to improve the appropriateness and speed of referral to severe asthma services and increase the number of patients benefiting from biologic treatments^14^. There have been no similar studies of the impact of asthma pathway changes published to date, although other work in the UK and internationally has shown that lack of adherence to guideline recommendations for asthma management is a barrier to effective service implementation^15–17^. There is also evidence of the potential benefits of training and education in primary care as a means of improving referrals to specialist services^18^, and of the impact of specialist assessment and management for severe asthma on patient and other outcomes^19,20^. The lack of capacity in specialist services has been recognised as contributing to sub-optimal management of patients with severe asthma, and recommendations on minimum service capacity, including MDTs, have been made by the UK Respiratory GIRFT (‘Getting It Right First Time’) report^21^. Our observed reductions in patient waiting times both overall and between discrete stages of the severe asthma pathway compare favourably with audit data reported in 2022^11^. Analysis of clinical data suggest that patient quality of life and symptom control were improved as a result of the enhanced severe asthma pathway, and health service use reduced also. These benefits have been observed in multiple studies^22–26^. It is possible that the enhanced severe asthma pathway was also associated with cost savings, although a cost analysis was outside the scope of this evaluation. However, establishing cost-effectiveness is important: reviews of the cost-effectiveness of asthma biologics highlight differences between different biologics, driven by the risk of anaphylaxis and suitability for self-administration; the degree to which they are targeted towards specific patient sub-groups, and whether drug costs are discounted^27,28^.

This study had some limitations. Changes to the severe asthma pathway took place in a single centre, and a comparative analysis of effectiveness in multiple centres would be required to ascertain whether the observed benefits to patients and services would be generalisable elsewhere. For the quantitative evaluation, patient data in the historical patient cohort for the standard pathway were limited, which must be borne in mind when interpreting the statistical findings relating to changes in pathway waiting times between the standard and enhanced pathways. Similarly, there were only 46 patients available for the complete case analysis of patient and other outcomes data. Mean follow-up after biologics initiation for these patients was only 6.3 months due to delays in implementing the enhanced pathway, so the impact of the pathway changes after the full 12 months initial implementation period could not be established. Qualitatively, fewer interviews were possible with participants from primary care than initially anticipated, which may impact on the reported barriers and facilitators to the enhanced asthma pathway within this setting. It was also not possible to recruit any staff working in the regional tertiary care severe asthma service, so this perspective is absent from our data. Finally, performing a cost analysis was outside the scope of this evaluation. Any such analysis would have to assess potential financial benefits against the full cost of operating the enhanced pathway, bearing in mind that resource savings may accrue to different parts of the healthcare system than those that bear the costs. This may have important implications for service commissioning.

Despite being a study of changes to the severe asthma pathway at a single centre, the improvements in processes and outcomes achieved by the study suggest several implications for policy and practice. Data showed that training and education of staff in primary care was instrumental in improving the number and appropriateness of patient referrals to the specialist service in secondary care, but that resource implications in all settings should be considered. It is likely that ongoing funding is required to ensure that the longer-term sustainability of the enhanced pathway is assured and is able to become fully embedded as routine practice. Importantly, establishing the sustainability of the linkages and relationships between primary and secondary care should be prioritised, so that primary care providers are able to identify and refer suitable patients to specialist services with less reliance on the nurse educator to perform this role on their behalf. The engagement of primary care with the enhanced pathway is essential to its ongoing success.

In conclusion, the implementation of an enhanced pathway for the identification and management of patients with severe asthma using asthma biologics was associated with substantial reductions in patient waiting times and significant reductions in prescribing rates, hospital admissions and improved asthma control. The large number of referrals to specialist asthma services generated from a comparatively small number of GP practices confirms a potentially large level of unmet need that an enhanced severe asthma can help to address. Ongoing work must ensure the cost-effectiveness and longer-term sustainability of the enhanced pathway within clinical practice.

## Methods

### Changes made to the severe asthma pathway

**Fig. 1:**
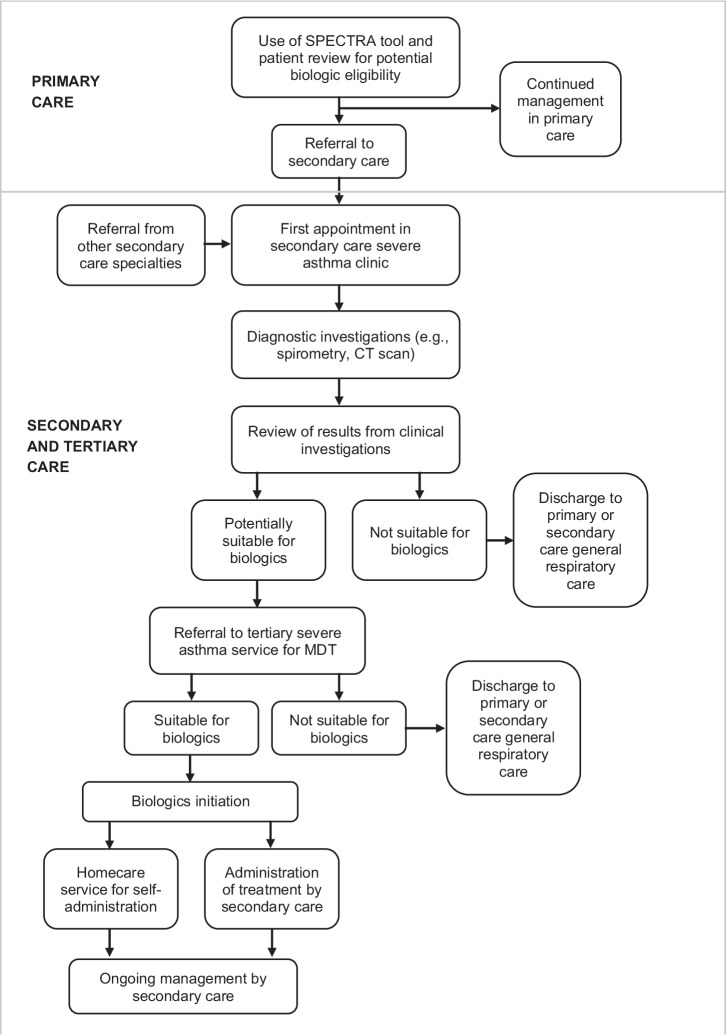
Enhanced patient pathway for severe asthma management.

The enhanced pathway aimed to initiate an additional 120 patients on asthma biologics over 12 months from November 2021 to November 2022. Pathway changes were implemented across primary, secondary and tertiary care, with a particular focus on optimising patient identification and referral from primary care to the secondary care severe asthma centre and reducing patient waiting times across the pathway (Table 4). The patient pathway is illustrated in Fig. 1. In primary care, the focus was on training and education for primary care practitioners, delivered by a nurse educator between May 2022 and October 2022. The nurse educator was a registered asthma nurse who had previously worked in community specialist respiratory services focusing on the management of complex respiratory conditions. She was trained to provide education to patients and service providers, having held a visiting lecturer position at a university in the West Midlands. Training content was designed to increase attendees’ knowledge and confidence in relation to multiple dimensions of severe asthma care: i) caring for patients with asthma; ii) managing patients with asthma; iii) stepping asthma treatment up or down; iv) confirming asthma diagnosis, and v) knowing when referral to secondary care specialist services is appropriate. Training materials included pre-existing resources (including clinical guidelines, resources developed by NHS England and Health Education England), and other materials developed by the nurse educator to create a short training session of around 90 min duration which could be tailored according to participants’ education needs. Training was delivered in multiple ways to maximise reach and engagement: delivered remotely or face-to-face in single sessions using a webinar/seminar delivery style (organised via the local Primary Care Network and Staffordshire Training Hub for GP training); via regional group events attended by primary care staff from multiple general practices, and on an individual general practice basis. By the time training delivery ceased, 187 healthcare professionals had participated in training, representing 37 primary care practices across Stoke and Staffordshire.

**Table 4:** Changes made to severe asthma pathway.

Setting	Area of change	Specific features of change
PRIMARY CARE	Patient identification	•Use of SPECTRA tool (AstraZeneca software for GPs which applies referral criteria to practice lists, identifies eligible patients and optimises referral to secondary care)
	Simplified referral form and patient management algorithm	•Based on successful pathway developed by Dudley Respiratory Group
	Training/education	•Using materials already available and tailored information developed specifically •Training delivered face-to-face or remotely to individual practices and via region-wide group events and webinars
SECONDARY CARE	Clinics and capacity	•Introduction of a biologics-specific multidisciplinary team •Severe asthma/biologics clinic •Increased pharmacy capacity
	Home care and self-administration	•Nurse advice •Online information/training •Self-administration training •Injection reminders and adherence trackers •Reports to clinical team re exacerbations
TERTIARY CARE	Clinics and capacity	•Increased multidisciplinary team capacity

The nurse educator also assisted a number of GP practices to identify patients who may be potentially eligible for biologic treatments by running the SPECTRA tool^29^ on their practice lists. This tool was developed by Astra Zeneca and is designed to identify patients with suspected severe asthma who may meet the criteria for biologic treatments (patients with more than one hospitalisation, intensive care unit (ICU) stay or mechanical ventilation in the past 12 months; two or more prescriptions for systemic corticosteroids in the previous six months; six or more reliever inhalers in the previous 12 months, and/or poor symptom control as shown by an Asthma Control Questionnaire (ACQ) score of less than 20). A ‘target list’ of GP practices was developed using data from the NHS Business Services Authority (NHSBSA) respiratory dashboard which identified the top 50 oral steroid and SABA prescribers in the UHNM catchment area. These GP practices were then approached by the nurse educator to participate in the SPECTRA element of the study, with 28 of the 50 agreeing to participate. At each participating practice, patients who were identified by the SPECTRA tool were reviewed individually by primary care staff and the nurse educator to decide whether or not they should be referred to the secondary care severe asthma clinic at UHNM. Patients who were not referred to secondary care continued to be managed in primary care and were asked to attend an annual asthma review at the practice. SPECTRA was run on each general practice’s system once, with SPECTRA and individual patient review taking between one and five hours depending on practice size. No further interventions were provided in primary care beyond training/education for primary care staff and patient identification/review following the use of the SPECTRA tool at each participating practice.

Once referred to the secondary care severe asthma clinic, patients received usual care with no specific interventions in the secondary or tertiary care setting beyond that provided routinely (e.g. diagnostic assessment and standard advice concerning treatment adherence and symptom control). Changes to the pathway in specialist asthma services focused on increasing staff capacity through the establishment of additional staff posts and the introduction of an additional biologics-specific MDT (increasing capacity to two clinics per week instead of one) to expedite patient assessment and reduce waiting times across the pathway. Pharmacy staff capacity was increased to facilitate timely treatment prescribing, and the existing home care service for biologics was expanded.

### Funding for the enhanced severe asthma pathway

All changes to the severe asthma pathway were funded by the grant from the NHS AAC programme (totalling £100k over 12 months). This funded the increased clinic capacity within secondary care, additional MDTs, increased pharmacy capacity and all additional staffing needs via contributions to existing staff work time equivalents or, in the case of the nurse educator and a part-time secondary care asthma nurse, creation of new fully-funded staff posts. Neither Astra Zeneca nor any other industry organisation provided any funding for the project either directly or via the NHS AAC, nor did they have access to the results of running the SPECTRA tool on general practice computer systems. All staff involved in delivering the enhanced pathway were employed by University Hospitals North Midlands.

### Evaluation design

The evaluation was undertaken by the Applied Research Collaboration (ARC) West Midlands, and used a convergent parallel mixed methods design^30^ combining quantitative analysis of routinely-collected process and outcomes data with qualitative, semi-structured interviews focusing on pathway implementation.

### Quantitative data collection

Routinely-collected, anonymised clinical data on processes and outcomes were obtained from University Hospitals North Midlands. Data covered asthma biologics use (number of patients initiating biologics during the project, number of patients using the home care service); prescribing (rates of steroid inhaler, SABA and OCS use); rates of hospital admission; number of referrals from primary care to specialist asthma services; process data (patient waiting times between specific points on the severe asthma pathway), and differences in asthma control scores measured using the ACQ at baseline and three months after commencing biologics. With 50 patients providing data before and after biologics treatment and a minimal clinically important difference of 0.5 points (SD 0.97)^31^ this would have more than 90% power to detect a difference of 0.5 points on the ACQ6.

### Quantitative data analysis

Trends in key metrics were analysed over time. Data on asthma biologics use and engagement with the home care service focused on the cohort of patients initiating biologics treatment during the project. Monthly rates of inhaled corticosteroids, SABA and OCS prescribing and hospital admissions were compared for each patient during the 12 months immediately before biologics initiation and up to 12 months post-initiation. ACQ6 scores were compared for each patient before biologics initiation and four months after initiation. Comparative analysis of patients’ pre- and post-biologics metrics were carried out on a complete case basis for patients initiating biologics treatment under the enhanced pathway who had at least 3 months follow-up data after biologics initiation, using paired t-tests (two-sided). Mean patient waiting times in weeks between each discrete stage of the severe asthma pathway and the overall time from primary care referral to biologics initiation were compared for a cohort of historic patients (*n* = 37) who were referred from primary care between November 2020 and November 2021 (i.e., prior to implementation of the enhanced pathway), against the cohort of patients referred from November 2021 onwards (*n* = 143) (i.e. under the enhanced pathway) using independent t-tests (two-sided). All statistical analyses were conducted using SPSS version 29 (2022; Armonk, NY: IBM Corp).

### Semi-structured interviews: eligibility and recruitment

Interviews were undertaken with stakeholders across primary, secondary and tertiary care (GPs, practice nurses, nurse educator, respiratory clinicians and key operational managers). Stakeholders were purposively sampled in collaboration with project leads at Royal Stoke University Hospital to ensure representation from a broad range of organisations involved in developing severe asthma care across the health economy. In addition to purposive sampling, each interviewee was asked to suggest additional key individuals for the evaluation team to approach, using snowballing to ensure all relevant perspectives were included. Up to 20 interviews were planned, with the final sample size guided by thematic saturation.

### Qualitative data collection

Interviews took place between July and November 2022 and were conducted over the telephone or via secure video conference software. Interviews were audio-recorded, and each lasted between 14 and 48 min. A pre-defined topic guide was followed, assessing participants’ views about how the enhanced severe asthma pathway worked in practice, perceptions of barriers and facilitators to effective implementation, and how challenges were overcome.

### Qualitative data analysis

Following each interview, the audio-recording was transcribed verbatim, and the transcript proof-read against the original audio file by the researcher who undertook the interview. Two members of the evaluation team analysed and independently coded at least 10% of the interview transcripts using NVivo, with results compared and discussed until agreement was reached. Data were analysed thematically^32^, and organised for interpretation using the key domains of the Consolidated Framework for Implementation Research (CFIR)^33^. The CFIR is a typology consisting of five domains, each containing a number of constructs that may influence the effectiveness of an intervention or service improvement initiative. This typology allowed us to understand how the enhanced severe asthma pathway was implemented, what worked where and why.

### Data synthesis

Quantitative and qualitative data were synthesised to develop recommendations for policy and practice about the most effective configuration of the severe asthma pathway.

### Inclusion and ethics

Ethical approval was obtained from the University of Birmingham Research Ethics Committee (Ref: ERN_22_0069) in March 2022 and from the Health Research Authority (HRA) in May 2022 (IRAS ID: 311869). Research governance approval was obtained from University Hospitals North Midlands (UHNM) in May 2022. Interview participants provided written informed consent. All methods used in this study were performed in accordance with the relevant ethical guidelines and regulations.

### Reporting summary

Further information on research design is available in the Nature Research Reporting Summary linked to this article.

### Supplementary information

Reporting summary

## Data Availability

The qualitative data that support the findings of this study are available on request from the corresponding author (SD) at s.l.damery@bham.ac.uk. The data are not publicly available as they contain information that could compromise the privacy of research participants. The clinical data were obtained from a third party (University Hospitals North Midlands) and cannot be made available.
