# Supplementary material for: Mixed-methods evaluation of an enhanced asthma biologics clinical pathway in the West Midlands UK
Source: NPJ Prim Care Respir Med. 2024 May 1;34:7. doi: 10.1038/s41533-024-00365-y (PMC11063170; doi:10.1038/s41533-024-00365-y)
Supplement: Supplementary file 1 — Reporting summary [file 41533_2024_365_MOESM1_ESM.pdf]

nature portfolio

Corresponding author(s): Dr Sarah Damery
Last updated by author(s): Jan 24, 2024



# Reporting Summary

Nature Portfolio wishes to improve the reproducibility of the work that we publish. This form provides structure for consistency and transparency in reporting. For further information on Nature Portfolio policies, see our Editorial Policies and the Editorial Policy Checklist.

## Statistics

For all statistical analyses, confirm that the following items are present in the figure legend, table legend, main text, or Methods section.

| n/a | Confirmed | |
|---|---|---|
| ☐ | ☒ | The exact sample size ($n$) for each experimental group/condition, given as a discrete number and unit of measurement |
| ☐ | ☒ | A statement on whether measurements were taken from distinct samples or whether the same sample was measured repeatedly |
| ☐ | ☒ | The statistical test(s) used AND whether they are one- or two-sided<br>*Only common tests should be described solely by name; describe more complex techniques in the Methods section.* |
| ☒ | ☐ | A description of all covariates tested |
| ☒ | ☐ | A description of any assumptions or corrections, such as tests of normality and adjustment for multiple comparisons |
| ☐ | ☒ | A full description of the statistical parameters including central tendency (e.g. means) or other basic estimates (e.g. regression coefficient) AND variation (e.g. standard deviation) or associated estimates of uncertainty (e.g. confidence intervals) |
| ☐ | ☒ | For null hypothesis testing, the test statistic (e.g. $F$, $t$, $r$) with confidence intervals, effect sizes, degrees of freedom and $P$ value noted<br>*Give P values as exact values whenever suitable.* |
| ☒ | ☐ | For Bayesian analysis, information on the choice of priors and Markov chain Monte Carlo settings |
| ☒ | ☐ | For hierarchical and complex designs, identification of the appropriate level for tests and full reporting of outcomes |
| ☒ | ☐ | Estimates of effect sizes (e.g. Cohen's $d$, Pearson's $r$), indicating how they were calculated |

*Our web collection on statistics for biologists contains articles on many of the points above.*

## Software and code

Policy information about availability of computer code

| | |
|---|---|
| Data collection | No code was used to collect data in this study |
| Data analysis | No code was used to analyse data in this study |

For manuscripts utilizing custom algorithms or software that are central to the research but not yet described in published literature, software must be made available to editors and reviewers. We strongly encourage code deposition in a community repository (e.g. GitHub). See the Nature Portfolio guidelines for submitting code & software for further information.

## Data

Policy information about availability of data

All manuscripts must include a data availability statement. This statement should provide the following information, where applicable:

- Accession codes, unique identifiers, or web links for publicly available datasets
- A description of any restrictions on data availability
- For clinical datasets or third party data, please ensure that the statement adheres to our policy

The qualitative data that support the findings of this study are available on request from the corresponding author (SD) at s.l.damery@bham.ac.uk. The data are not publicly available as they contain information that could compromise the privacy of research participants. The clinical data were obtained from a third party (University Hospitals North Midlands) and cannot be made available.

# Research involving human participants, their data, or biological material

Policy information about studies with human participants or human data. See also policy information about sex, gender (identity/presentation), and sexual orientation and race, ethnicity and racism.

| | |
|---|---|
| Reporting on sex and gender | Consent was not provided for sharing of individual-level data on characteristics of patients who were assessed for biologic treatment eligibility. Sex of these patients is therefore not reported in the manuscript. We do not report the sex of individuals who participated in the qualitative element of the evaluation. |
| Reporting on race, ethnicity, or other socially relevant groupings | No assignment of patients to groups on the basis of any social, cultural or economic groupings was made in any of the analyses for this study. No sub-group analysis was performed on the data regarding patient's engagement with the asthma biologic treatment pathway. Interview participants are described by their job role and the setting in which they worked (primary care, secondary care) only. |
| Population characteristics | See above |
| Recruitment | This study reports an evaluation of changes to a clinical pathway for access to asthma biologics treatments. Patients who went through the pathway were not recruited; they were identified by their general practitioner in collaboration with the asthma educator working on this study. Patients who were identified as being high users of short-acting beta agonists or who had had multiple asthma exacerbations etc. were assessed by the asthma educator to determine whether they may benefit from a referral to specialist secondary care asthma services. Thus there was not 'recruitment' process for the asthma patients. For the qualitative interviews, individuals from primary care, secondary care and tertiary care were purposively sampled to ensure representation from a broad range of organisations involved in developing severe asthma care across the health economy. Each individual who participated in interviews was also asked to identify (through snowballing) any other relevant individuals who should be approached to participate. There may be self-selection bias in interview participation which means that those who are more enthusiastic about the asthma pathway or the study may be more likely to participate in an interview. However, as participation in research is voluntary, we could not compel individuals to take part in interviews. The potential bias is noted in our manuscript: first, fewer interviews were undertaken with participants from primary care than initially anticipated, which may impact on the reported barriers and facilitators to the enhanced asthma pathway within this setting. It was also not possible to recruit any staff working in the regional tertiary care severe asthma service, so this perspective is absent from our data. |
| Ethics oversight | Ethical approval was obtained from the University of Birmingham Research Ethics Committee (Ref: ERN_22_0069) in March 2022 and from the Health Research Authority (HRA) in May 2022 (IRAS ID: 311869). Research governance approval was obtained from University Hospitals of North Midlands (UHNM) in May 2022. Interview participants provided written informed consent. All methods used in this study were performed in accordance with the relevant ethical guidelines and regulations. |

Note that full information on the approval of the study protocol must also be provided in the manuscript.

# Field-specific reporting

Please select the one below that is the best fit for your research. If you are not sure, read the appropriate sections before making your selection.

☒ Life sciences ☐ Behavioural & social sciences ☐ Ecological, evolutionary & environmental sciences

For a reference copy of the document with all sections, see nature.com/documents/nr-reporting-summary-flat.pdf

# Life sciences study design

All studies must disclose on these points even when the disclosure is negative.

| | |
|---|---|
| Sample size | Routinely-collected, anonymised clinical data on processes and outcomes were obtained from University Hospitals of North Midlands. Data covered asthma biologics use (number of patients initiating biologics during the project, number of patients using the home care service); prescribing (rates of steroid inhaler, SABA and OCS use); rates of hospital admission; number of referrals from primary care to specialist asthma services; process data (patient waiting times between specific points on the severe asthma pathway), and differences in asthma control scores measured using the ACQ at baseline and three months after commencing biologics. With 50 patients providing data before and after biologics treatment and a minimal clinically important difference of 0.5 points (SD 0.97) this would have more than 90% power to detect a difference of 0.5 points on the ACQ6. |
| Data exclusions | There were no exclusions of data - this was an evaluation of data collected by University Hospitals North Midlands on patient progression through the severe asthma pathway. The only exclusions were for the comparison made in clinical outcomes between baseline and study end, for which only patients who had commenced biologic treatments during the study period were included (complete case analysis). |
| Replication | The findings for this study cannot be replicated in the sense that this was a real world evaluation of a clinical service. We have provided as much detail as is reasonable about the way that the severe asthma pathway was changed in order to improve its effectiveness in managing patients with severe asthma by expanding and expediting their potential access to effective asthma biologic treatments. |
| Randomization | No randomisation was undertaken, there was only a single group in the study. |
| Blinding | Blinding was not possible/appropriate as there was only a single study group. |

# Reporting for specific materials, systems and methods

We require information from authors about some types of materials, experimental systems and methods used in many studies. Here, indicate whether each material, system or method listed is relevant to your study. If you are not sure if a list item applies to your research, read the appropriate section before selecting a response.

## Materials & experimental systems

| n/a | Involved in the study |
| --- | --- |
| ☒ | ☐ Antibodies |
| ☒ | ☐ Eukaryotic cell lines |
| ☒ | ☐ Palaeontology and archaeology |
| ☒ | ☐ Animals and other organisms |
| ☒ | ☐ Clinical data |
| ☒ | ☐ Dual use research of concern |
| ☒ | ☐ Plants |

## Methods

| n/a | Involved in the study |
| --- | --- |
| ☒ | ☐ ChIP-seq |
| ☒ | ☐ Flow cytometry |
| ☒ | ☐ MRI-based neuroimaging |

## Plants

| | |
| --- | --- |
| Seed stocks | *Report on the source of all seed stocks or other plant material used. If applicable, state the seed stock centre and catalogue number. If plant specimens were collected from the field, describe the collection location, date and sampling procedures.* |
| Novel plant genotypes | *Describe the methods by which all novel plant genotypes were produced. This includes those generated by transgenic approaches, gene editing, chemical/radiation-based mutagenesis and hybridization. For transgenic lines, describe the transformation method, the number of independent lines analyzed and the generation upon which experiments were performed. For gene-edited lines, describe the editor used, the endogenous sequence targeted for editing, the targeting guide RNA sequence (if applicable) and how the editor was applied.* |
| Authentication | *Describe any authentication procedures for each seed stock used or novel genotype generated. Describe any experiments used to assess the effect of a mutation and, where applicable, how potential secondary effects (e.g. second site T-DNA insertions, mosiacism, off-target gene editing) were examined.* |
